# Hydrochlorothiazide-Induced Exfoliative Rash and Sepsis

**DOI:** 10.7759/cureus.63655

**Published:** 2024-07-02

**Authors:** Krishna Sheth, Cody Lee, Neil Srinivas, Yusif Hasan, Khin Myat

**Affiliations:** 1 Internal Medicine, Garnet Health Medical Center, Middletown, USA; 2 Internal Medicine, Touro College of Osteopathic Medicine, Middletown, USA

**Keywords:** thiazide diuretics, diuretics, sepsis, exfoliative rash, hydrochlorothiazide

## Abstract

Hydrochlorothiazide is a diuretic agent commonly used to treat hypertension, chronic edema from congestive heart failure or cirrhosis, and nephrogenic diabetes insipidus. Diuretics work by increasing urine output and are classified based on the specific renal segments they act on. As with all medications, they are not without their side effects. The most significant and serious include hypovolemia and electrolyte disturbances. Other more rare adverse effects include dermatitis and hypersensitivity reactions. We discuss an 86-year-old male who presented to the emergency room with complaints of lightheadedness and progressive exfoliating rash that began shortly after starting hydrochlorothiazide in an outpatient setting.

## Introduction

Thiazide diuretics, such as hydrochlorothiazide, are frequently used to treat hypertension or peripheral edema. They inhibit sodium-chloride cotransporters on the apical surface membrane in the distal convoluted tubule (DCT). Around 5%-10% of renal sodium absorption occurs at the DCT [1]. The increased fraction of sodium remaining in the interstitial lumen enables more free water to enter and be excreted. Adverse effects are common and are usually related to electrolyte abnormalities or renal dysfunction. This includes hypokalemia, hyponatremia, hypercalcemia, metabolic alkalosis, and hyperglycemia [2]. In some instances, patients with a sulfonamide drug allergy may display cross-reactivity and hypersensitivity with thiazide diuretics due to their common sulfa structures [3]. Clinical presentations are similar to classic allergic responses such as rash, anaphylaxis, and eosinophilia. Hydrochlorothiazide-induced drug rash is a very rare side effect of thiazide diuretics [4].

## Case presentation

**Figure 1:**
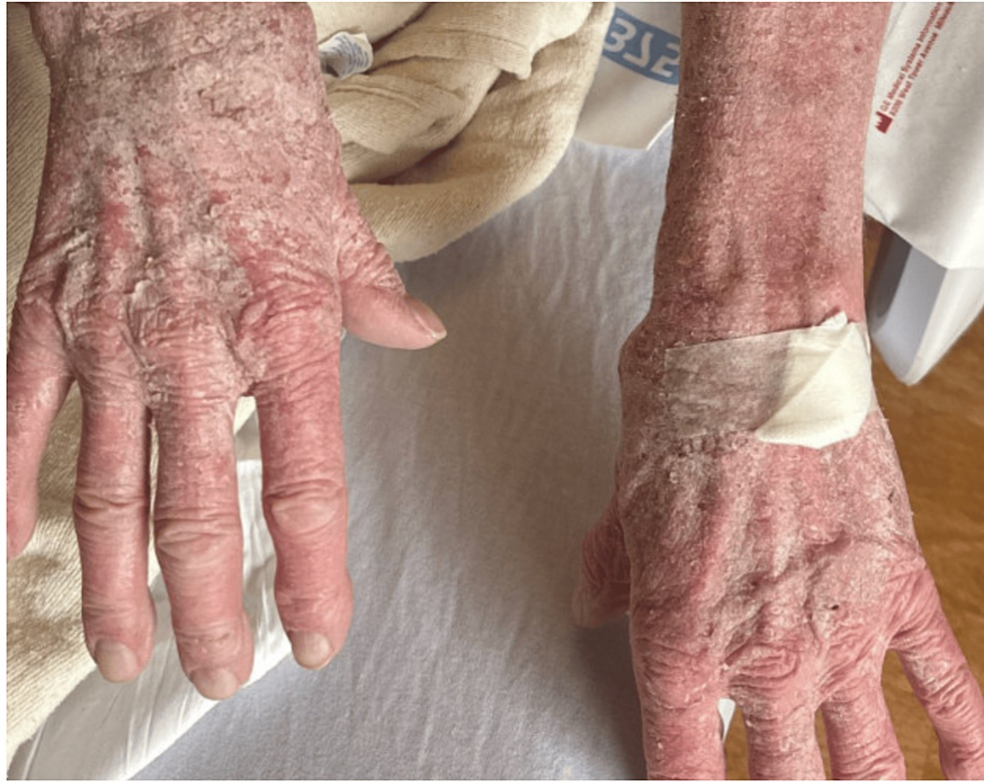
Exfoliative rash on bilateral arms

An 86-year-old male with a past medical history of symptomatic bradycardia with a permanent pacemaker, heart failure with preserved ejection fraction, non-insulin-dependent diabetes mellitus, benign prostate hypertrophy, asthma, emphysema, hypothyroidism, and dementia presented to the emergency department (ED) with a diffuse, progressively worsening generalized rash and lightheadedness that began one month ago. It was reported that the rash began on his hands and progressed to his upper body. The rash was extremely pruritic and showed no improvement after topical steroid use. He was started on hydrochlorothiazide and carvedilol one month prior to arrival for cardioprotection after permanent pacemaker placement. Other medications include mirtazapine, atorvastatin, donepezil, memantine, metformin, and Stiolto and albuterol inhalers. Pertinent social history included former smoking. In the emergency department (ED), the patient was afebrile and severely hypotensive (65/39 mmHg). Physical examination findings showed a diffuse exfoliative rash on the chest, back, face, neck, and arms, as seen in Figure 1.

**Figure 2:**
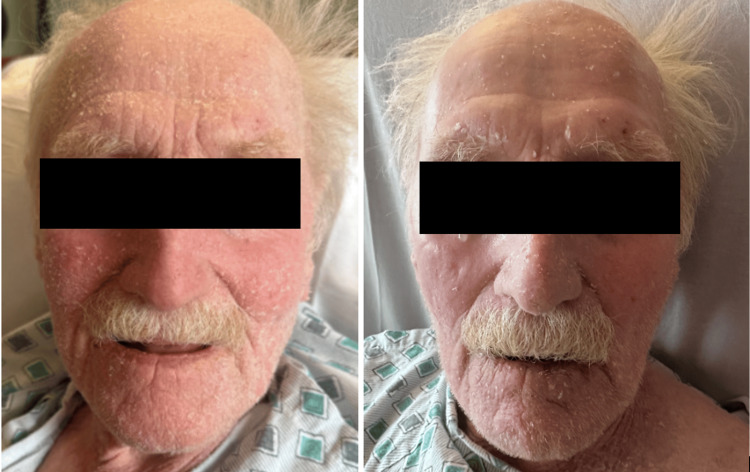
Exfoliative rash before stopping hydrochlorothiazide (left) and after stopping hydrochlorothiazide (right)

Laboratory results were significant for a leukocytosis of 23,900/mm^3^, lactic acid of 2.9 mmol/L, creatinine of 1.97 mg/dL that was increased from baseline of about 1.34 mg/dL, hyponatremia, and marked eosinophilia. Chest X-ray was unremarkable, and no infiltrates or consolidations were seen. He was started on intravenous (IV) fluids and IV corticosteroids. Despite adequate fluid resuscitation in the ED, the patient had a fever with a maximum temperature of 104.5°F and significant leukocytosis. The patient was admitted for sepsis of unclear source. In the ED, hydrochlorothiazide was discontinued. Several days later, his suspected drug-induced rash showed improvement after discontinuation of hydrochlorothiazide and intravenous corticosteroids, as seen in Figure 2. 

Further sepsis workup revealed an elevated procalcitonin 54.743 ng/mL and elevated lactic acid. Four out of four blood cultures obtained grew *Staphylococcus aureus*. Empiric antibiotic therapy was started with intravenous vancomycin and ceftriaxone. The hospital course was complicated by new-onset atrial fibrillation (AF) with rapid ventricular response (RVR) and worsening respiratory status. Infectious disease and cardiology were consulted. Per infectious disease, the patient had severe sepsis secondary to high-grade *S. aureus* bacteremia with suspected translocation from skin defects caused by exfoliative rash. Vancomycin was continued, and ceftriaxone was switched to cefazolin. An echocardiogram was also recommended to rule out endocarditis or vegetative disruption of the pacemaker in lieu of new-onset AF with RVR. Per the cardiology consult, the patient was continued on Eliquis for anticoagulation and metoprolol for rate control.

## Discussion

Thiazide-associated dermatitis usually presents with a diffuse maculopapular rash within 1-2 weeks of starting the medication. Associated fever may also be present. The dermatologic manifestations are due to sulfonamide cross-reactivity and drug-induced photosensitivity. Thiazide diuretics frequently cause photosensitivity reactions, with a prevalence of 1-100 per 100,000 patients [5]. The literature is extremely limited on thiazide-induced dermatitis, although it is a commonly used medication.

In this case, the patient presented with a progressively worsening and diffuse exfoliative rash one month after starting hydrochlorothiazide for blood pressure control, thus leading to a high clinical suspicion of drug-induced dermatitis. The rash improved with the discontinuation of hydrochlorothiazide, intravenous corticosteroids, and local skin care. However, other more serious consequences ensued even after cessation of hydrochlorothiazide, such as septicemia secondary to translocation of bacteria through the bacteria left in the skin.

## Conclusions

Thiazide diuretics are commonly used in the management of primary hypertension and peripheral edema. They are generally considered safe and effective; however, clinicians must remain vigilant of potential adverse effects, especially with the elderly and those with comorbidities. Cases of thiazide diuretic dermatitis should be identified promptly within the clinical context so that the offending agent may be discontinued. Documented cases of thiazide-associated dermatitis are minimal; therefore, a proper history and timeline should be conducted to eliminate it as a potential cause.
